# Ingleby Lectures : Lecture IV

**Published:** 1891-05-02

**Authors:** 


### SURGERY.

### INGLEBY LECTURES.—No. IV.

Mr. Jordan Lloyd gave his fourth lecture on the subject of "Hernia":—

Hernias are very common in babies, especially amongst the hospital class of people. Inguinal hernia is by far the most common variety; umbilical is frequently met with, but the femoral variety is very rarely seen.

Hernia is met with in babies of every condition of development—the mature, the immature, the puny, and the strong. It is sometimes met with at birth, but more often during the first few weeks of life. Boys are more often affected than girls.

Many factors have to do with the causation of inguinal hernia, such as any condition giving use to straining, as, for instance, phimosis, intestinal catarrh, &c., but Mr. Lloyd considers the chief predisposing cause to be the way in which the belly-binder is applied to the newly-born infant, a stout, inelastic piece of calico being several times firmly wound round the belly, thereby squeezing the infantile bowels with undue force against the abdominal walls, the popular idea being that babies must be bound firmly to prevent their bellies spreading. Hernias in children are reducible unless strangulated, and never contain omentum. Small intestine usually occupies the sac, occasionally the cæcum descends into it. As for treatment, the majority of cases are easily controlled, and cured by simple mechanical means. The simplest method is to employ the "Worsted Truss"—an appliance but too little known. You merely take a skein of worsted and fasten one portion round the waist, and pass another portion from the circular band in front over the hernia, between the legs to the circular band again behind, thereby using what amount of pressure you like. If this fails to restrain the hernia, then some form of the old spring truss must be employed, taking care that the spring is not too strong, and that it exerts its pressure over the right place, which is over the internal ring and inguinal canal. If the hernia cannot be kept reduced by one of these methods, then its radical cure by operation should be undertaken, for the radical cure of hernia by modern methods is a very safe procedure; and surely it is better to cure the deformity than to allow the patient to struggle on through life in constant danger of strangulation.

As regards the radical cure of hernia, Mr. Lloyd stated that the operation most suitable for young children is simple excision of the sac. In performing this operation the few following points are worthy of notice: (i.) Do not first reduce the hernia, for the fuller your sac the easier it is exposed, and with a capable surgeon there should be no danger of damaging the gut inside. (ii.) In cutting down on the sac, the several layers are best divided by simply cutting down on them without using a ground director at all. (iii.) Get close down on to the sac before you begin to separate it from its surroundings. Remember in children the cremasteric fascia forms a distinct and well-marked layer of muscle; beneath it is the infundibuliform fascia, also well marked. You will know the sac, because it has no blood vessels on it. (iv.) Strip the sac from its surroundings by the finger nail, or handle of the scalpel. When completely separated, pull it down firmly; ligature its neck as close as possible to the external ring, and then divide the sac below, but not nearer than half an inch to the ligature. (v.) In the majority of cases it is not necessary to suture the pillars of the ring, especially in children under three. In older children, if the opening admits the finger tip, it may be necessary. (vi.) In the congenital variety, *i.e.*, when the hernia and testis are occupying the same cavity, the operation is more difficult, for after dividing the neck of the sac it is still attached at the rete testis. You can now either leave a portion so as to form an artificial tunica vaginalis, or you can cut it through close to the gland itself. (vii.) After the operation a pad and bandage or worsted truss should be worn five or six weeks, and may then be discarded. Mr. Lloyd quoted 25 cases of boys on whom he had operated as above under seven years of age. 24 were done by open incision, 14 without, and 10 with pillar suture. All these made good recoveries. One he did subcutaneously, and this patient died—he believed from secondary shock.

*Strangulated Hernia* in children is comparatively rare. Of its symptoms the most important is "plus tension," *i.e.*, when the tension of the sac wall is greater than that of the belly wall. A tense hernia with a softer abdomen means strangulation, and demands immediate relief. A tense hernia rapidly becoming soft means gangrene or rupture of the bowel.

Strangulated hernias in children call for earlier operation and more gentle taxis than in the adult. Herniotomy in children is a less dangerous operation if it is supplemented by the operation already described for radical cure. A hernia into the sac of an undescended testicle should be treated by excision of the sac, and the gland should be removed.

*Hydrocele.*—This, again, is a very common condition in young children. All hydroceles are transparent, and so are easily diagnosed from herniæ. They are often cured by small patches of iodine on the scrotum, or by simple friction. If they persist, Mr. Lloyd says he has abandoned the old-fashioned ways of treatment, viz.: Injection of irritating fluids, or the introduction of a seton. He recommends excision of the sac, an operation very similar to that for the radical cure of hernia. The operation varies somewhat according to the kind of hydrocele. Thus, in (i.) *The Vaginal Hydrocele*, simple dilatation of the tunica vaginalis, you treat by excision close up to the tritum of the testicle. (ii.) *Infantile Hydrocele*, distension of the unobliterated funicular process as high as the inguinal rings: this you treat as in the first variety. (iii.) *Congenital Hydrocele*, distension of an unobliterated funicular process which freely communicates with the peritoneal cavity: here you ligature the neck of the sac, and then remove it, as in the last variety. (iv.) *Funicular Hydrocele*, distension of the funicular process which is cut off below from the tunica vaginalis testis: here you operate just as you do for hernia, you ligature the neck of the sac, and excise it. (v.) *Encysted Hydrocele of Cord*, distension of a portion only of the funicular process, which is separate both from the peritoneal cavity and the tunica vaginalis: here you merely cut down on the sac and shell it out of its bed. In these operations it is important to ligature every bleeding point, so as to keep clear of a subsequent hæmatocele forming.

